# Acknowledgement to Reviewers of *Journal of Fungi* in 2019

**DOI:** 10.3390/jof6010017

**Published:** 2020-01-21

**Authors:** Journal of Fungi Editorial Office

**Affiliations:** MDPI, St. Alban-Anlage 66, 4052 Basel, Switzerland; jof@mdpi.com

The editorial team greatly appreciates the reviewers who have dedicated their considerable time and expertise to the journal’s rigorous editorial process over the past 12 months, regardless of whether the papers are finally published or not. In 2019, a total of 116 papers were published in the journal, with a median time to first decision of 38 days and a median time from submission to publication of 18 days. The editors would like to express their sincere gratitude to the following reviewers for their generous contribution in 2019:
Abdolrasouli, AlirezaLee, Chao-HungAgrawal, Samir G.Lee, Soo ChanAimanianda, Vishu KumarLegrand, MélanieAlberti, FabrizioLeitão, AnaAllison, Devon LeaLevesque, EricAllsopp, Luke P.Li, ShaojieAlonso-Monge, RebecaLi, TaoTaoAlspaugh, J. AndrewLi, Xiu-MeiAlves, VivianeLin, Chi-JanAmiri, AchourLindsley, Mark D.Antinori, SpinelloLitvintseva, Anastasia P.Araniciu, CătălinLombardi, LisaArendrup, MaikenLopez-Ribot, JoseAtkin, Audrey L.Lorenz, AlexanderBahr, Nathan C.Lundy, Fionnuala TBal, Abhijit M.Lutterloh, EmilyBaldin, ClaraLyman, MeghanBarker, Bridget M.Maddi, AbhiramBecker, PierreMalacrinò, AntoninoBedre, ReneshMantadakis, ElpisBenny, GeraldMarkinson, BryanBernal, PatriciaMarques, ClaudiaBernal-Martínez, LeticiaMartinez, LuisBicanic, TihanaMcClelland, Erin E.Bleackley, Mark R.Meya, David B.Boelker, MichaelMillon, LaurenceBorman, Andrew M.Mora-Montes, HectorBraissant, O.Moran, GaryBretagne, StéphaneMorio, FlorentBrinkmann, VolkerMourad, AhmadBrun, SylvainMouyna, IsabelleBuil, Jochem B.Mukaremera, LilianeBuitrago, María JoséMunoz, JoseCai, LeiNacher, MathieuCalderone, RichardNakayama, HaruoCampuzano, AltheaNanjappa, Som G.Candon, SophieNarra, Hema PrasadCannon, RichardNobbs, AngelaCaro, YanisNosanchuk, JoshuaChamilos, GeorgiosNowrousian, MinouChander, JagdishOniga, Smaranda DafinaChauhan, NeerajOvery, David P.Chen, Yee-ChunÖzenci, VolkanChen, Zhi-YuanPagano, MarcelaChilders, Delma SusanPark, Young-JinCoelho, CarolinaPasqualoto, Alessandro C.Coleman, Jeffrey J.Pawłowska, JuliaColombo, ArnaldoPedrosa, Ana FilipaCortegiani, AndreaPerochon, AlexandreCorzo-Leon, DoraPeters, BrandilynCorzo-León, Dora E.Peters, BrianCrum-Cianflone, Nancy FPetraitis, VidmantasCulotta, ValeriaPfaller, Michael A.Datta, KausikPires, Regina HelenaDavari, AgrinPonce De León, AlfredoDavis, JamesPonikau, Jens U.Dawson, Thomas L.Popolo, Laura MariaDe Groot, Piet W. J.Portilla, MaribelDe Hoog, SybrenPosch, WilfriedDeepe, GeorgePrigione, ValeriaDesai, JigarQueiroz-Telles, FlavioDiamond, GillRamachandiran, IyappanDrogari-Apiranthitou, MariaRangel, Drauzio E.N.Drummond, Rebecca A.Rapala-Kozik, MariaDufossé, LaurentRappleye, ChadDymarska, MonikaReed, Kevin F.M.Edgerton, MiraRichardson, RiinaElad, DanielRobert-Gangneux, FlorenceErdman, ScottRobinson, SeriEsher, ShannonRomo, JesusEspinel-Ingroff, AnaRostislav, ZemekFranco, ChrisRotstein, ColemanFrealle, EmilieRupp, SteffenFuchs, BethSabino, RaquelGago, SaraSamaranayake, LakshmanGambino, AlessioSampaio, PaulaGandía, MónicaSchmidt, MartinGarcia-Diaz, JuliaSchöps, RicardoGarcia-Sherman, MelissaSchwarz, PatrickGarre, VictorianoSegal, EstherGelli, AngieSellam, AdnaneGonzalez, GabrielSexton, Joseph D.Grabinska, KarionaSharma, CheshtaGroll, AndreasSharp, JoGuitard, JulietteShaw, Brian D.H. Caceres, DiegoShi, MeiqingHage, ChadiShoham, ShmuelHagen, FerrySilva, Sónia CarinaHalliday, CatrionaSimonetti, GiovannaHamill, RichardSipsas, Nikolaos V.Haneke, EckartSobrado, PabloHao, GuixiaSolano, FranciscoHarro, JanetteStaniszewska, MonikaHay, RodStchigel, Alberto MiguelHendrickx, MarijkeStevens, DavidHennequin, ChristopheSun, Man-HongHennicke, FlorianSwidergall, MarcHillmann, FalkTalbot, NickHoenigl, MartinTaylor-Smith, LeanneHong, Chang PyoTeixeira, Marcus MIkeda, KenichiThekkiniath, JoseJackson, Brendan RThielen, BethJacobsen, IlseTorović, LjiljaJohnson, ElizabethTorren, Laura VilanovaJohnston, SimonUzman, DenizJunqueira, Juliana CamposValdes, AudreyKasanen, RistoVallabhaneni, SnigdhaKauffman, CarolVargas, SergioKavanagh, KevinVautier, SimonKean, RyanVlahovic, TraceyKidd, Sarah E.Voss, AndreasKirkland, Theo N.Wang, ZheKirschner, RolandWarris, AdiliaKitagaki, HiroshiWelsh, RoryKong, Won-SikWengenack, NancyKordalewska, MilenaWhite, LewisKovacs, RenatoWickes, Brian L.Krom, BastianWilliamson, Peter R.Kubicek, ChristianWimmer, ReinhardKucharíková, SoňaWöstemeyer, JohannesKumar, PradeepWurster, SebastianKumar, RohitashwXu, JianpingKumaresan, PappanaickenXu, JintaoLachance, Marc-AndréXue, ChaoyangLackner, MichaelaYan, Dong-HuiLake, Douglas F.Yun, YingziLanternier, FannyZaias, NardoLarriba, GermanZhao, YananLavergne, Rose-Anne


